# Best Evidence Summary for the Management of Frailty in Elderly Patients in Emergency Department–Integrative Review

**DOI:** 10.1002/nop2.70720

**Published:** 2026-07-29

**Authors:** Meiling Yuan, Haipeng Yang, Yueguang Dai, Xiaotong Sun, Yuchen Zhang, Xia Liu

**Affiliations:** ^1^ Emergency Department Affiliated Hospital of Qingdao University Qingdao People's Republic of China; ^2^ Intensive Care Unit Affiliated Hospital of Qingdao University Qingdao People's Republic of China; ^3^ Department of Nursing Affiliated Hospital of Qingdao University Qingdao People's Republic of China

**Keywords:** best evidence/evidence‐based nursing, emergency, frailty, geriatric

## Abstract

**Aim:**

To systematically search, evaluate and synthesize the most robust evidence regarding the management of frailty in elderly patients in the emergency department.

**Design:**

This study was conducted as an evidence summary, strictly adhering to the reporting standards for evidence summaries developed by the Center for Evidence‐Based Nursing, Fudan University.

**Data Sources:**

UpToDate, BMJ Best Practice, JBI, National Guideline Clearing‐house, Guidelines International Network, Scottish Intercollegiate Guidelines Network, National Institute for Health and Care Excellence, Registered Nurses Association of Ontario, Yi Mai tong Guidelines Network, the Cochrane Library, PubMed, Web of Science, Embase, OVID, Sinomed, CNKI, Wan Fang database. The search was conducted from the inception of each database to June 2025.

**Review Methods:**

Evidence was selected using predefined eligibility criteria, critically appraised with AGREE II, AMSTAR 2, and Joanna Briggs Institute tools as appropriate, and synthesized using the JBI evidence classification and recommendation system. Study selection, appraisal and evidence extraction were undertaken independently by trained reviewers, with disagreements resolved through discussion or third‐reviewer adjudication.

**Results:**

After rigorous screening and evaluation, 13 high‐quality articles were identified, including 3 clinical guidelines, 1 randomized controlled trial (RCT), 2 clinical decision‐making studies, 2 expert consensus documents, 2 cohort studies and 3 systematic reviews. Through collaborative in‐depth deliberation, 34 evidence‐based insights on emergency geriatric frailty management were extracted, synthesized, and categorized into 10 key themes.

**Conclusion:**

Healthcare professionals in emergency departments should prioritize geriatric frailty and select the highest‐quality evidence tailored to clinical realities. This approach will enhance the standardization and systematicity of geriatric frailty management in emergency settings.

**Impact:**

Our study provides a structured approach (i.e., a stratified frailty assessment and intervention pathway, and a nurse‐led multidisciplinary emergency frailty management model) and novel practical management methods (including an integrated screening‐assessment‐intervention‐discharge continuous care protocol and an age‐friendly emergency environment optimization strategy) for managing geriatric frailty in emergency settings, and promotes nursing services tailored to older adults. Collectively, these contributions aim to address the challenges of managing geriatric frailty in emergency departments and enhance the effectiveness of nursing interventions.

**Reporting Method:**

This research followed the evidence summary reporting specifications of the Fudan University Center for Evidence‐based Nursing.

**Trial Registration:**

The registration number is ‘ES20257380’.

**For the Professional Field and Patient Care Implications:**

The findings provide emergency nurses with actionable guidance for triage‐based frailty screening, escalation to comprehensive assessment, medication reconciliation, mobility and nutrition support, caregiver engagement and structured discharge handover.

**Patient or Public Contribution:**

No patients or members of the public were involved because this study synthesized previously published evidence.

## 1 Introduction

Population aging is accelerating worldwide, and increasing numbers of older adults live with multimorbidity and geriatric syndromes. Emergency departments are a major point of access for acute and critical care in this population, where high attendance, complex care needs, resource use and prolonged length of stay challenge health systems internationally (Mooijaart et al. 2021). Geriatric emergencies include sudden illness, accidental injury, and acute exacerbation of chronic disease in older adults (American College of Emergency Physicians et al. 2014).

Frailty is a distinct geriatric syndrome characterized by reduced physiological reserves, which increases vulnerability and impairs stress resilience—rendering even minor external stressors capable of precipitating adverse clinical events (Birong 2014). Consequently, frail older adults frequently present to EDs due to such stressors (Shang and Guo 2022). An exclusive focus on emergency symptom management, to the neglect of frailty intervention, elevates adverse outcomes, including higher readmission rates, increased mortality and prolonged hospital stays (Afilalo et al. 2020). Frailty affects 9.7%–43.7% of older adults presenting to EDs (Afilalo et al. 2020; Qinyi et al. 2023). Importantly, timely intervention and preventive strategies can effectively ameliorate or even reverse frailty, reduce adverse outcomes and prevent 3%–5% of deaths in this population (Shamliyan et al. 2013).

Although frailty is a chronic, progressive syndrome, an acute ED presentation can trigger or accelerate functional decline. The ED is therefore a critical point for early identification, risk mitigation, and referral. However, the nursing‐specific evidence remains fragmented: screening tools, escalation criteria, nurse‐led interventions, caregiver engagement, discharge communication, and follow‐up processes are not consistently integrated into a single operational pathway. This gap limits nurses' ability to translate frailty evidence into routine triage, assessment, care coordination and transition practices. The present review therefore synthesized the best available evidence to define practical, evidence‐based nursing actions for frail older adults in EDs and to support standardized, nurse‐led multidisciplinary care.

## 2 Methods/Methodology

Structured evidence‐based questions were developed using the PIPOST model, a standardized tool widely used in evidence‐based nursing research for constructing evidence‐based problems. This model can clearly define the core elements of research and ensure the comprehensiveness and accuracy of evidence retrieval. Given its suitability for designing evidence summary research on clinical nursing management, the PIPOST model was selected to construct the evidence‐based questions of this study (Zhu et al. 2017) (Evidence‐Based Nursing Center, Fudan University). Specifically, the components of this model applied in the study were defined as follows: (P, Population) older adults aged ≥ 60 years presenting to the emergency department (ED); (I, Intervention) frailty screening (rapid identification of frailty/pre‐frailty status), frailty assessment (comprehensive evaluation of frailty severity, aetiology and risk factors), frailty management (multidisciplinary intervention, medication optimization, exercise guidance, nutritional support, discharge planning and continuous care); (P, Professional) ED healthcare professionals; (O, Outcome) key outcomes, including patient mortality, revisit rate and readmission rate; (S, Setting) ED (as the setting for implementing evidence‐based practices); and (T, Type of Evidence) types of evidence, including clinical decision‐making tools, clinical practice guidelines, evidence summaries, systematic reviews, expert consensus and RCTs.

### 2.1 Literature Retrieval Method

To systematically collect guidelines, expert consensus statements, original documents, evidence summaries and systematic reviews related to frailty management in elderly ED patients, a comprehensive search was conducted across prominent domestic and international databases. Guided by the 6S model (Dicenso et al. 2009), three researchers independently retrieved relevant literature from BMJ Best Practice, UpToDate, National Institute for Health and Care Excellence (NICE), Registered Nurses' Association of Ontario (RNAO), Scottish Intercollegiate Guidelines Network (SIGN), Guidelines International Network (GIN), Yimaitong, Joanna Briggs Institute (JBI) Evidence‐Based Healthcare Center, Cochrane Library, PubMed, Web of Science, CINAHL, China National Knowledge Infrastructure (CNKI), Wanfang Database, SinoMed and China Science and Technology Journal Database (VIP). The search period covered from the inception of each database to June 2025, with a combined strategy of Medical Subject Headings (MeSH) and free‐text terms adopted to ensure comprehensive coverage. A librarian was consulted during the development of the search strategy to optimize its precision and effectiveness. Our specific search terms and strategies for major databases are presented in Supporting Information: Materials.

### 2.2 Literature Screening

The inclusion criteria of this study were defined as follows: (1) Research subjects were elderly patients (≥ 60 years) admitted to the ED; (2) Studies focused on the management of frail elderly patients in the ED; (3) Eligible literature types included clinical practice guidelines, evidence summaries, systematic reviews, expert consensus statements and RCTs not covered by the aforementioned evidence‐based resources; (4) Literature was published in Chinese or English; (5) For revised or updated guidelines, only the latest version was included; (6) The intervention and observation of frail elderly patients in the included studies were completed in the ED or closely linked to ED management.

The exclusion criteria were defined as follows: (1) Abstracts, conference papers and research proposals were excluded; (2) Papers with inaccessible full texts were excluded; (3) Republished papers or updated versions of previously included papers were excluded; (4) Papers failing to meet the predefined quality evaluation criteria were excluded.

### 2.3 Evaluation of Literature Quality

The quality of clinical practice guidelines was evaluated using the 2017 updated Appraisal of Guidelines for Research and Evaluation II (AGREE II) (Zhou, Hao, et al. 2018). This comprehensive instrument comprises 23 items across six domains and two overarching assessment criteria. Each item was scored on a 1–7 Likert scale, with higher scores indicating stronger agreement. We categorized the final recommendation grades into three tiers: Grade A (a score exceeding 60% across all six domains), Grade B (a score of 30%–60% in three or more domains) and Grade C (a score below 30% in three or more domains).

The Assessment of Multiple Systematic Reviews 2 (Zhang et al. 2018), a systematic assessment tool, was used to evaluate the systematic reviews included in this study. This tool comprises 16 items, 7 of which are critical items (Items 2, 4, 7, 9, 11, 13, 15). Studies with no non‐compliant non‐critical items or only one non‐compliant non‐critical item were categorized as high quality; those with one or more non‐compliant non‐critical items as medium quality; those with only one non‐compliant critical item as low quality; and those with more than one non‐compliant critical item as very low quality.

Quai‐experimental study (Zhou, Gu, et al. 2018a), expert consensus statements (Zhu et al. 2020) and RCT (Zhou, Gu, et al. 2018b) were each assessed using the relevant tools developed by the JBI.

Two researchers with systematic training in evidence‐based methodology independently conducted a comprehensive assessment of literature quality and collaboratively deliberated on the assessment results. In cases of disagreement, authoritative experts specializing in evidence‐based research were consulted for adjudication. When conflicting evidence emerged from different sources, priority was given to evidence consistent with established principles, derived from high‐quality studies and recently published.

### 2.4 Summary and Classification of Evidence

Two researchers independently extracted relevant information from the included literature using content extraction methods, with a third researcher responsible for verifying and integrating the extracted data. The following extraction principles were applied: (1) When duplicate evidence was identified, priority was given to that which conforms to professional terminology and is readily comprehensible. (2) Complementary evidence was merged into a single evidence body based on logical associations. (3) For conflicting evidence, the principle of prioritizing evidence‐based, high‐quality and the latest authoritative evidence was observed. This study employed the JBI Evidence Pre‐Classification and Evidence Recommendation Levels System (Wang and Hu 2015) for evidence classification and recommendation grading, which categorizes evidence into Levels 1–5. Evidence recommendation levels (Level A: strong recommendation; Level B: weak recommendation) were jointly determined by two researchers independently based on evidence level, feasibility, appropriateness, clinical significance, validity and other relevant factors, and the consistency test was conducted to ensure the reliability of the grading process; in case of disagreement, the third researcher was consulted for decision.

## 3 Result

### 3.1 Search Results

**FIGURE 1:**
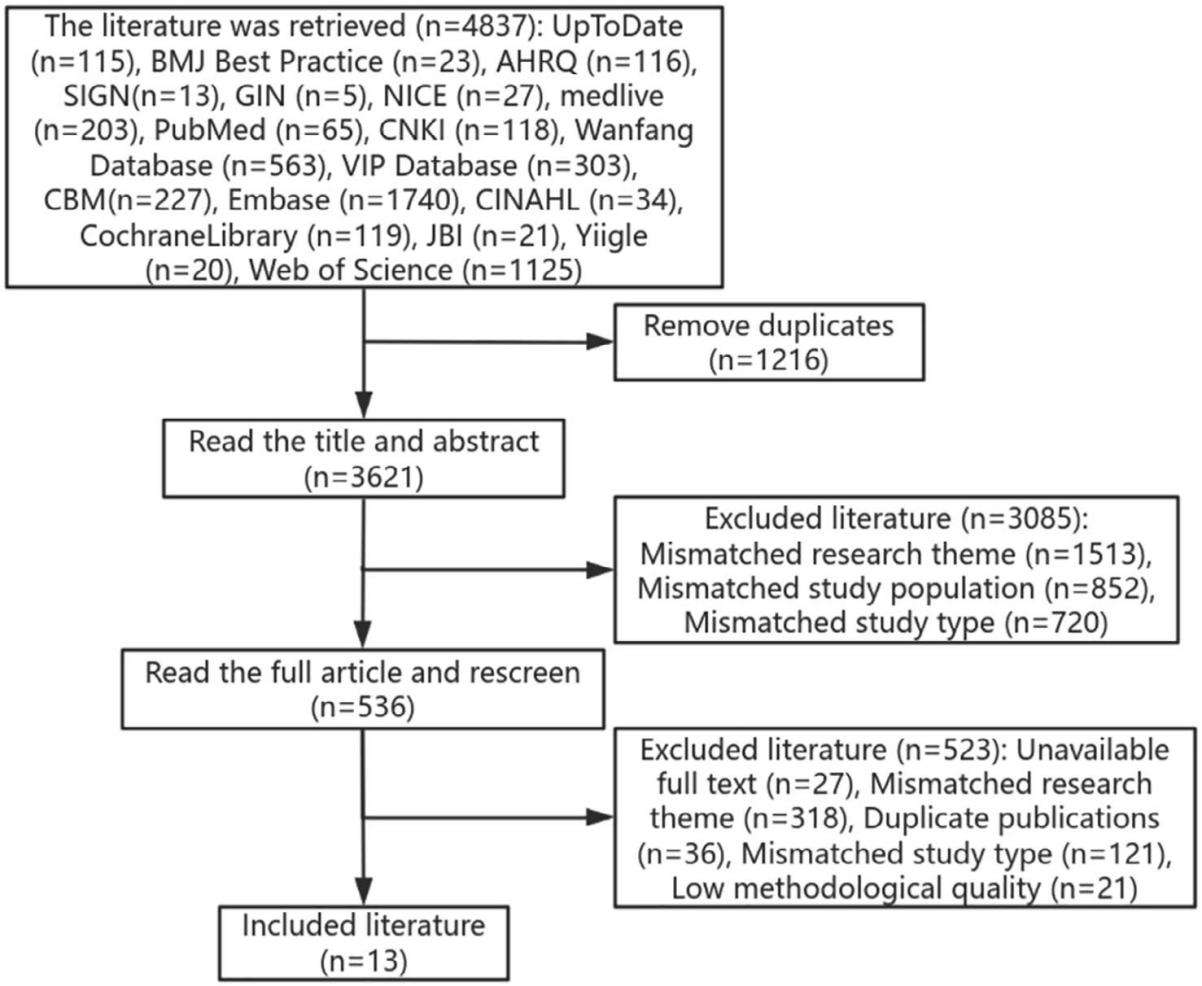
Flow chart of literature screening.

A total of 4837 studies were initially retrieved. Following sequential screening, 13 studies were ultimately included, specifically 2 on clinical decisions (Reuben 2018; Walston 2018), 3 clinical practice guidelines (Dent et al. 2017, 2019; Health, M 2023), 3 systematic reviews (Apóstolo et al. 2018; Arakelyan et al. 2023; Ellis et al. 2011), 2 expert consensuses (Geriatric Medicine Branch of Chinese Medical Association 2022; Geriatric Medicine Branch of Chinese Medical Association et al. 2017), 1 RCT (Ekerstad et al. 2017) and 2 quasi‐experimental studies (Shrapnel et al. 2019; Wallis et al. 2018). The literature screening process is illustrated in Figure 1, and the general characteristics of the included studies are summarized in Table 1.

**TABLE 1:** Basic characteristics of included studies (*n* = 13).

Authors	Source	Year of publication	Type of literature	The literature theme
Walston (2018)	UpToDate	2018	Clinical decision	Frailty
Reuben (2018)	UpToDate	2018	Clinical decision	Office‐based assessment of the older adult
Dent et al. (2017)	PubMed	2017	Guideline	The Asia‐Pacific Clinical Practice Guidelines for the Management of Frailty
Health, M (2023)	Medlive	2023	Guideline	Frailty in Older Adults ‐ Early Identification and Management
Dent et al. (2019)	PubMed	2019	Guideline	Physical Frailty:ICFSR International Clinical Practice Guidelines for Identification and Management
Apóstolo et al. (2018)	PubMed	2018	Systematic review	Effectiveness of interventions to prevent pre‐frailty and frailty progression in older adults: a systematic review
Arakelyan et al. (2023)	PubMed	2023	Systematic review	Effectiveness of holistic assessment‐based interventions for adults with multiple long‐term conditions and frailty: an umbrella review of systematic reviews
Ellis et al. (2011)	BMJ	2011	Systematic review	Comprehensive geriatric assessment for older adults admitted to hospital: meta‐analysis of randomisedcontrolled trials
Geriatric Medicine Branch of Chinese Medical Association et al. (2017)	Yiigle	2017	Expert consensus	Chinese experts consensus on assessment and intervention for elderly patients with frailty
Geriatric Medicine Branch of Chinese Medical Association (2022)	Yiigle	2022	Expert consensus	Chinese expert consensus on prevention of frailty in the elderly (2022)
Ekerstad et al. (2017)	Web of Science	2017	RCT	Acute care of severely frail elderly patients in a CgA‐unit is associated with less functional decline than conventional acute care
Shrapnel et al. (2019)	Embase	2019	Quai‐experimental study	A nurse‐led model of care within an emergency department reduces representation rates for frail aged care residents
Wallis et al. (2018)	Web of Science	2015	Quai‐experimental study	The Geriatric Emergency Department Intervention model of care: a pragmatic trial

### 3.2 Literature Quality Evaluation Results

#### 3.2.1 Clinical Decisions

The two clinical decisions (Reuben 2018; Walston 2018) are obtained from UpToDate, indicating strong evidence.

#### 3.2.2 Quality Evaluation Results of Guidelines

Three guidelines (Dent et al. 2017, 2019; Health, M 2023) were incorporated into the present study. Specifically, two of these guidelines (Dent et al. 2019; Health, M 2023) achieved a score of ≥ 60% across all domains, with a recommendation level of Grade A, whereas one (Dent et al. 2017) was rated Grade B. The methodological quality assessment results of these guidelines are presented in Table 2.

**TABLE 2:** Quality evaluation of included guidelines (*n* = 3).

Guidelines	Standardized scores in various domains (%)						≥ 60%	≥ 30%	ICC	Quality evaluation
	Scope and purpose	Stakeholder involvement	Rigour of development	Clarity of presentation	Applicability	Editorial independence				
Health, M (2023)	76.19	76.19	77.67	71.42	76.78	67.85	6	6	0.973	A
Dent et al. (2019)	85.71	88.09	92.85	85.71	83.92	82.14	6	6	0.994	A
Dent et al. (2017)	76.19	78.57	77.67	76.19	58.92	78.57	5	6	0.986	B

#### 3.2.3 Quality Evaluation Results of Systematic Reviews

Three systematic reviews (Apóstolo et al. 2018; Arakelyan et al. 2023; Ellis et al. 2011) were included in the present study, and their quality assessment results are summarized in Table 3. As indicated in this table, all three reviews achieved a quality evaluation outcome of ‘no or only one non‐critical entry was inconsistent’; thus, they were deemed to be of high quality and included in the analysis.

**TABLE 3:** Quality appraisal of systematic reviews (*n* = 3).

Items	Apóstolo et al. (2018)	Arakelyan et al. (2023)	Ellis et al. (2011)
1. Did the research questions and inclusion criteria for the review include the components of PICO?	Yes	Yes	Yes
2. Did the report of the review contain an explicit statement that the review methods were established prior to the conduct of the review and did the report justify any significantdeviations from the protocol?	Yes	Yes	Yes
3. Did the review authors explain their selection of the study designs for inclusion in the review?	Yes	Yes	Yes
4. Did the review authors use a comprehensive literature search strategy?	Yes	Yes	Yes
5. Did the review authors perform study selection in duplicate?	Yes	Yes	Yes
6. Did the review authors perform data extraction in duplicate?	Yes	Yes	Yes
7. Did the review authors provide a list of excluded studies and justify the exclusions?	Yes	Yes	Yes
8. Did the review authors describe the included studies in adequate detail?	Yes	Yes	Yes
9. Did the review authors use a satisfactory technique for assessing the risk of bias (RoB) in individual studies that were included in the review?	Yes	Yes	Yes
10. Did the review authors report on the sources of funding for the studies included in the review?	Yes	Yes	Yes
11. If meta‐analysis was performed, did the review authors use appropriate methods for statistical combination of results?	Yes	Yes	Yes
12. If meta‐analysis was performed, did the review authors assess the potential impact of RoB in individual studies on the results of the meta analysis or other evidence synthesis?	Yes	Yes	Yes
13. Did the review authors account for RoB in primary studies when interpreting/discussing the results of the review?	Yes	Yes	Yes
14. Did the review authors provide a satisfactory explanation for, and discussion of, any heterogeneity observed in the results of the review?	Yes	Yes	Yes
15. If they performed quantitative synthesis did the review authors carry out an adequate investigation of publication bias (small study bias) and discuss its likely impact on the results of the review?	Yes	Yes	Yes
16. Did the review authors report any potential sources of conflict of interest, including any funding they received for conducting the review?	Yes	Yes	Yes

#### 3.2.4 Quality Evaluation Results of Expert Consensus

Two expert consensus statements (Geriatric Medicine Branch of Chinese Medical Association 2022; Geriatric Medicine Branch of Chinese Medical Association et al. 2017) were included in the present study. Specifically, Item 6 of these statements was rated ‘NO’, while the remaining items were rated ‘YES’. All expert consensus statements were deemed to be of high quality and thus included, with details presented in Table 4.

**TABLE 4:** Quality appraisal of expert consensus statements (*n* = 2).

Items	Geriatric Medicine Branch of Chinese Medical Association et al. (2017)	Geriatric Medicine Branch of Chinese Medical Association (2022)
1. Whether the source of the opinion is clearly labelled?	Yes	Yes
2. Whether the views are derived from influential experts in the field?	Yes	Yes
3. Whether the views presented centred on the population interests relevant to the study?	Yes	Yes
4. Is the conclusion of the statement based on the results of the analysis? Is the presentation of ideas logical?	Yes	Yes
5. Are there references to other existing literature?	Yes	Yes
6. Whether there are inconsistencies between the ideas presented and previous literature?	No	No

#### 3.2.5 Quality Evaluation Result of the Randomized Controlled Trials

A single RCT (Ekerstad et al. 2017) was included in the present study. This included literature exhibited a rigorous structure and high methodological quality, with its quality assessment results summarized in Table 5.

**TABLE 5:** Quality evaluation of randomized controlled trials (*n* = 1).

Items	Ekerstad et al. (2017)
1. Whether the study subjects were actually randomized?	Yes
2. Whether allocation concealment has been achieved?	Yes
3. Whether the baseline was comparable between groups?	Yes
4. Whether study subjects were blinded?	Yes
5. Whether the intervener was blinded?	No
6. Whether outcome assessors were blinded?	Yes
7. Whether the groups receive the same measures other than the intervention to be validated?	Yes
8. Whether follow‐up was complete and, if not, whether measures were taken to manage loss to follow‐up?	Yes
9. Whether all randomly assigned subjects were included in the outcome analysis?	Yes
10. Whether the outcome indicators of each group were evaluated in the same way?	Yes
11. Whether the measurement methods of outcome indicators credible?	Yes
12. Adequacy of data analysis methods?	Yes
13. Was the study design sound? Is there anything different from the standard RCT in the conduct of the study and data analysis?	Yes

#### 3.2.6 Quality Evaluation Result of the Quai‐Experimental Studies

Two quasi‐experimental studies (Shrapnel et al. 2019; Wallis et al. 2018) were included in the present study. Both studies received an ‘unclear’ rating for Item 6: ‘Was follow‐up complete? If not, was loss to follow‐up reported and measures taken?’ All other items were rated ‘yes’. These studies were deemed well‐designed and of high quality, and thus included. Their quality assessment results are summarized in Table 6.

**TABLE 6:** Quality evaluation of quasi‐experimental studies (*n* = 2).

Items	Shrapnel et al. (2019)	Wallis et al. (2018)
1. Is the causal relationship in the study clearly explained?	Yes	Yes
2. Are the baseline conditions between groups comparable?	Yes	Yes
3. Are the other measures received by each group the same as the intervention to be validated?	Yes	Yes
4. Have you established a control group?	Yes	Yes
5. Were outcome measures measured in multiple dimensions before and after the intervention?	Yes	Yes
6. Was the follow‐up complete? If not, was the loss of follow‐up reported and were measures taken to address it?	Unclear	Unclear
7. Are the same methods used to measure the outcome indicators for each group of study subjects?	Yes	Yes
8. Are the methods used to measure outcome indicators reliable?	Yes	Yes
9. Is the data analysis method appropriate?	Yes	Yes

### 3.3 Summary and Description of Evidence

Through evidence extraction and synthesis, 34 pieces of evidence related to the emergency management of geriatric frailty were identified, covering 10 dimensions: necessity of frailty management, establishment of a multidisciplinary management team, frailty screening, frailty assessment, identification of risk factors, development of individualized frailty management plans, medication management, exercise and nutrition management, discharge planning and continuous improvement. These findings are presented in Table 7.

**TABLE 7:** Evidence summary of emergency geriatric wasting management.

Subject	Description of evidence	Level	Recommendation level
Necessity of frailty management	1. Frailty is a common age‐associated geriatric syndrome characterized by a decline in physiological reserve and increased vulnerability to internal and external stressors, which elevates the risks of falls, disability and mortality (Geriatric Medicine Branch of Chinese Medical Association 2022; Walston 2018)	3	B
	2. In the emergency department, a nurse‐led care model can alleviate frailty in elderly patients and is more cost‐effective than conventional medical care (G. Ellis et al. 2011; Shrapnel et al. 2019; Wallis et al. 2018; Walston 2018)	1	A
Establishment of a multidisciplinary management team	3. Establishing an emergency nurse‐led multidisciplinary team is recommended to coordinate the assessment and development of comprehensive care plans for frail older adults. This core team typically comprises emergency department nurses, emergency physicians, geriatricians (or clinicians with geriatric certification), clinical pharmacists, registered dietitians and rehabilitation specialists (i.e., occupational therapists and physical therapists) (Ekerstad et al. 2017; Health, M 2023; Walston 2018)	1	A
Frailty screening	4. Frailty screening is recommended to be performed by healthcare practitioners with specialized training in this domain, including geriatricians, primary care physicians, nurses, medical specialists, and dedicated medical staff (Dent et al. 2019)	1	A
	5. It is recommended that standardized rapid frailty screening tools be integrated into routine clinical practice for the emergency care of elderly patients aged 65 years and above. The selection of such tools should be guided by the characteristics of individual medical institutions and specific clinical contexts, with priority accorded to validated, evidence‐based assessment instruments (Dent et al. 2019)	3	A
	6. For ED elderly patients with unexplained weight loss exceeding 5% within 1 year, validated frailty assessment tools are recommended to evaluate frailty (Dent et al. 2017; Health, M 2023; Walston 2018)	2	A
	7. Early identification of frailty among elderly patients transferred from nursing homes to emergency departments is recommended (Dent et al. 2019; Shrapnel et al. 2019)	1	A
	8. The FRAIL Frailty Scale, Physical Frailty Phenotype, Vulnerable Elders Survey‐13 (VES‐13), Study of Osteoporotic Fractures (SOF) Index, and Edmonton Frailty Scale are rapid and effective screening tools for frailty in older adults, among which FRAIL Scale is recommended for integration into routine medical history‐taking for frailty assessment in ED (Geriatric Medicine Branch of Chinese Medical Association 2022; Reuben 2018; Walston 2018)	4	A
	9. Step rate is an important indicator for evaluating the mobility and physical function of elderly patients in the ED, which is closely related to the occurrence of frailty and falls. Therefore, it is recommended to include step rate and fall history in the routine frailty assessment of elderly patients in the ED (Reuben 2018).	3	A
Frailty assessment	10. Routine frailty assessment is not recommended for the general population of older adults; for ED elderly patients, targeted frailty assessment is only performed for those with positive screening results, which is in line with ED clinical resource allocation characteristics (Dent et al. 2017, 2019; Health, M 2023; Walston 2018)	1	A
	11. Older adult patients with frailty screening results indicative of frailty or pre‐frailty are recommended to complete frailty assessment for diagnostic purposes (Dent et al. 2019; Shrapnel et al. 2019)	1	A
	12. Frailty assessment should commence with medical history collection (Reuben 2018)	3	A
	13. Validated tools are recommended for frailty assessment, among older adult patients with recurrent hospital admissions, frailty may be identified through medical record review (Arakelyan et al. 2023; Dent et al. 2017; Ekerstad et al. 2017; Health, M 2023)	1	A
	14. When diagnosing clinical asthenia, clinicians should systematically formulate a differential diagnosis checklist to comprehensively screen for underlying physical illnesses and psychosomatic disorders presenting with asthenia‐related manifestations (Dent et al. 2017; Shrapnel et al. 2019)	2	A
Identification of risk factors	15. Frailty development is influenced by multiple factors: (1) Physiological factors: involuntary weight loss (particularly ≥ 5 kg over the prior year), urinary incontinence, sensory impairment (visual or hearing deficits) and chronic pain; (2) Clinical factors: polypharmacy; delirium, cognitive dysfunction or dementia; recurrent emergency department visits or hospitalizations; reduced functional status with mobility impairments; recent falls; heightened sensitivity to medication adverse effects; and inadequate social support (Dent et al. 2017; Ekerstad et al. 2017; Health, M 2023; Walston 2018)	1	B
	16. Identifying treatable frailty risk factors in ED elderly patients may prevent frailty progression (Dent et al. 2017)	1	A
Development of individualized frailty management plans	17. Person‐centered frailty management is an effective intervention for optimizing frailty‐related outcomes across hospitalized, emergency, and outpatient populations (Apóstolo et al. 2018; Arakelyan et al. 2023; Geriatric Medicine Branch of Chinese Medical Association 2022)	1	A
	18. A patient‐centered comprehensive assessment should be conducted, including: identifying the patient's core needs; reviewing their established treatment goals, values, and personal preferences; thoroughly examining the medical history, current disease status, and implemented interventions; initiating a medication review when indicated; and engaging in detailed communication and care coordination with the patient and their caregiver regarding follow‐up care plans and discharge planning (Dent et al. 2017; Ekerstad et al. 2017; Health, M 2023)	4	B
	19. Advanced care management plans should be developed for patients with frailty or in the pre‐frailty state, including polypharmacy optimization, sarcopenia‐specific interventions, identification of treatable causes of unintentional weight loss and fatigue, and targeted preventive and rehabilitative strategies to slow, prevent, or reverse frailty‐related functional decline (Dent et al. 2019; Geriatric Medicine Branch of Chinese Medical Association 2022; Geriatric Medicine Branch of Chinese Medical Association et al. 2017; Health, M 2023; Reuben 2018; Walston 2018)	1	A
	20. A patient‐centered shared decision‐making model should be established to facilitate multidimensional communication among healthcare providers, patients, and caregivers (Dent et al. 2017; Walston 2018)	2	A
	21. Frailty management is adaptively optimized through ongoing assessment of frailty‐related functional status and individualized treatment goals (Health, M 2023; Reuben 2018; Walston 2018)	2	B
	22. The Comprehensive Geriatric Assessment (CGA) not only guides the development of individualized frailty‐specific intervention plans but also informs the design of targeted risk‐mitigation strategies by identifying modifiable frailty‐related risk factors (Dent et al. 2017; Shrapnel et al. 2019; Walston 2018)	1	A
	23. During emergency department care, family members should be actively engaged in the clinical decision‐making and care of older adults with frailty (Arakelyan et al. 2023; Dent et al. 2017, 2019; Health, M 2023)	1	A
Development of individualized frailty management plans	24. Routine pharmacotherapy is not recommended as a first‐line intervention for the management of frailty (Dent et al. 2019; Geriatric Medicine Branch of Chinese Medical Association 2022; Geriatric Medicine Branch of Chinese Medical Association et al. 2017)	5	b
Medication management	25. Multidisciplinary teams should partner with older adults and their primary caregivers to implement evidence‐based, frailty‐adapted multimodal medication management strategies. These strategies include systematic medication reconciliation, dosage optimization, therapeutic drug monitoring, and dynamic frailty‐focused assessment—all aimed at enhancing medication safety and treatment adherence (Arakelyan et al. 2023; Ekerstad et al. 2017; Geriatric Medicine Branch of Chinese Medical Association 2022; Health, M 2023; Shrapnel et al. 2019; Walston 2018)	4	A
	26. Inappropriate or unnecessary prescriptions should be targeted for gradual reduction or discontinuation to mitigate polypharmacy among older adults with frailty (Dent et al. 2017; Geriatric Medicine Branch of Chinese Medical Association 2022).	1	A
Exercise and nutrition management	27. Exercise regimens should be individualized and tailored to the physical function and mobility of older adults with frailty in ED, with low‐to‐moderate intensity physical activities incorporating progressive resistance training under professional guidance; nutrition therapists should provide targeted oral nutritional or protein‐energy supplementation as needed, screen for reversible causes of acute frailty and non‐intentional weight loss, and give high‐quality protein‐energy supplementation and vitamin D supplementation for vitamin D‐deficient patients (Dent et al. 2019; Geriatric Medicine Branch of Chinese Medical Association 2022; Geriatric Medicine Branch of Chinese Medical Association et al. 2017; Health, M 2023; Reuben 2018; Walston 2018)	1	A
Discharge planning	28. Individualized practical lifestyle modification advice—with particular attention to low‐to‐moderate intensity resistance training and adequate protein intake—should be provided to older adults with frailty to support functional resilience (Dent et al. 2017)	4	B
	29. Targeted family health education should be conducted to prevent the progression of pre‐frailty and frailty among older adults, supporting their functional independence (Apóstolo et al. 2018; Dent et al. 2017, 2019; Health, M 2023)	1	B
	30. For older adults with frailty who do not require emergency observation or hospitalization, multidisciplinary teams can support primary care providers in delivering essential care services (e.g., wound management, catheter replacement). Concurrently, these teams can coordinate community healthcare resources to establish a family‐community‐hospital care linkage mechanism—an approach that fully mobilizes patients' family support systems, ensures robust discharge planning, and facilitates seamless care continuity tailored to individual needs (Wallis et al. 2018; Walston 2018)	2	B
	31. When older adults with acute frailty are admitted to the emergency department (ED) or transferred to lower‐level medical institutions, emergency healthcare providers should communicate all current and anticipated nursing needs with ward or community care teams (including family physicians) during handover. This process should generate a multidisciplinary emergency frailty management summary (written and/or oral) that includes an ‘Advanced Nursing Practice Plan’ resource allocation details (e.g., medications, medication charts), and relevant support documentation to ensure care continuity (Wallis et al. 2018; Walston 2018)	2	B
Discharge planning	32. Targeted social support should be provided to older adults with frailty in EDs based on identified unmet care needs, and these individuals should be encouraged to adhere to personalized comprehensive management plans to optimize functional outcomes (Dent et al. 2019)	4	b
Continuous improvement	33. Continuous, targeted staff development programs should be implemented for emergency department (ED) personnel, and specialized training courses should be developed to standardize evidence‐based geriatric frailty management protocols (Arakelyan et al. 2023; Wallis et al. 2018)	1	A
	34. EDs should redesign ward environments to accommodate the needs of older adults—including creating age‐friendly, safety‐focused settings tailored to older adults with frailty—to enhance comfort and reduce fall risk (Ellis et al. 2011)	1	A

Regarding the necessity of frailty management, two pieces of evidence were retrieved, one of which is Level 1 evidence. This Level 1 evidence indicates that a nurse‐led care model can enhance the effectiveness of frailty management and achieve cost‐efficiency. For the establishment of a multidisciplinary management team, one piece of Level 1 evidence recommends forming an emergency nurse‐led multidisciplinary team to coordinate assessments and develop comprehensive care plans. Concerning frailty screening, seven pieces of evidence were obtained, two of which are Level 1 evidence. These Level 1 evidence emphasize that healthcare professionals trained in screening should conduct timely screening for elderly patients transferred from nursing homes to emergency departments. For frailty assessment, five pieces of evidence were identified, three of which are Level 1 evidence. These evidence suggest that validated tools should only be used for elderly patients with positive frailty screening results, while frequent hospital admissions can be evaluated through medical history reviews. Regarding the identification of risk factors, two pieces of Level 1 evidence recommend early identification to prevent the progression of frailty. For the development of individualized frailty management plans, nine pieces of evidence were retrieved, four of which are Level 1 evidence. These evidence propose that intervention plans should be guided by comprehensive geriatric assessments and involve family members in care participation.

Concerning medication management, two pieces of evidence were obtained, one of which is Level 1 evidence. This evidence recommends addressing polypharmacy by reducing inappropriate medications. For exercise and nutrition management, one piece of Level 1 evidence suggests formulating exercise plans tailored to patients' actual physical conditions and recommends screening for reversible causes and providing nutritional supplementation for patients with involuntary weight loss. For discharge planning, five pieces of evidence were retrieved, including one piece of Level 1 evidence. This evidence recommends home‐based health education to prevent the onset of potential frailty and the progression of existing frailty. Finally, two pieces of evidence (both Level 1) were identified for continuous improvement, recommending training programs for emergency department staff and modifications to ward environments to meet the needs of older adults.

## 4 Discussion

### 4.1 The Evidence Presented in This Study Is Both Scientifically Rigorous and Practically Applicable

This study strictly adhered to evidence‐based methodologies, encompassing comprehensive evidence retrieval, literature quality assessment, and evidence synthesis to generate an integrated evidence summary. Thirteen studies were included, which synthesized 34 pieces of evidence across 10 domains—including the clinical necessity of geriatric frailty management in emergency settings and the formation of multidisciplinary teams. These findings provide a robust reference for clinical nursing practice. Specifically, the results enable clinical nurses to clearly master validated geriatric frailty screening tools and management protocols tailored to emergency care contexts. Furthermore, the findings offer evidence‐based guidance for developing standardized and systematic geriatric frailty management protocols in emergency settings and underpin evidence‐informed clinical nursing practice.

### 4.2 Emphasis on the Management of Frailty in Elderly Patients in the Emergency Department and Early Implementation of Frailty Management Led by Emergency Nurses

With the escalating global population aging, frailty has emerged as a critical challenge in public health and global healthcare that warrants urgent attention. Therefore, it is essential to conceptualize frailty as a holistic phenomenon and adopt a multifactorial, comprehensive intervention approach (Kolle et al. 2023). Existing research (WANG Qinyi et al. 2023) indicates that the incidence of frailty among elderly patients in global emergency departments remains relatively high. However, the current management of frailty in emergency settings globally is still in the early exploratory stage, with multiple barriers in clinical practice—including insufficient knowledge among emergency healthcare providers, inefficient multidisciplinary collaboration mechanisms and the absence of standardized management protocols (Shang and Guo 2022). Multiple studies (Ekerstad et al. 2017; Shrapnel et al. 2019; Wallis et al. 2018) have confirmed that an emergency collaborative care model led by emergency nurses under physician supervision can significantly improve clinical outcomes: it effectively shortens the observation and hospitalization durations of elderly patients, reduces hospitalization costs and does not increase mortality or readmission rates for the same aetiology. The Multidisciplinary Acute Care for Elderly in the Emergency Department (MACIAE) model, developed by Shrapnel et al. (2019), establishes an in‐hospital multi‐departmental collaboration network and inter‐institutional linkage mechanisms with external medical institutions through emergency nurse‐led specialized case management strategies. This model enables seamless transitions between medical systems and in‐hospital nursing services for frail elderly patients presenting to the emergency department.

### 4.3 Screening for Frailty Constitutes the Initial Step in the Management of Elderly Patients Presenting With Weakness in the Emergency Department

Research on emergency care models (Sinha et al. 2011) clearly demonstrates that integrating frailty screening into routine triage and assessment workflows is a critical measure for the early identification and targeted intervention of high‐risk older adults. Studies (Geriatric Medicine Branch of Chinese Medical Association et al. 2017; Shang and Guo 2022) further emphasize the need to clarify the clinical positioning of frailty screening and assessment during implementation.

Current clinical practice employs multiple frailty assessment tools, which exhibit significant disparities in classification accuracy and prognostic validity. International guidelines (Dent et al. 2017) recommend that clinical institutions prioritize three key criteria when selecting frailty screening tools: (1) validity and reliability validated through large‐sample studies; (2) alignment of operational procedures with clinical practice requirements; (3) adaptability to the resource allocation characteristics of specific clinical settings.

Based on evidence‐based data, this study proposes a stratified frailty assessment strategy for older adults in emergency departments (Karam et al. 2015): emergency healthcare providers with systematic training should use standardized tools to conduct rapid frailty screening for patients aged ≥ 65 years (Dent et al. 2019). Notably, multiple studies (Dent et al. 2017; Ellis et al. 2022; Walston 2018) highlight that adult emergency patients with unintentional weight loss exceeding 5 kg within 1 year should also be prioritized for screening.

For patients identified as frail or pre‐frail, comprehensive geriatric assessments—encompassing potential etiological diagnosis and standardized evaluation of frailty severity—are recommended. Emergency departments should select appropriate screening tools based on patient flow and demographic characteristics; for older adults with high visit frequencies, establishing an electronic medical record tagging system to dynamically monitor frailty status is suggested to optimize screening efficiency.

### 4.4 Providing Individualized and Continuous Frailty Management for Elderly Patients in Emergency Care Settings Is Essential to Improving Their Frailty Status

The management strategies discussed in this section are all based on the clinical characteristics of elderly patients in the ED (acute onset, limited treatment time, complex comorbidities), and the interventions are targeted to meet the actual needs of ED clinical practice. The Expert Consensus on Assessment and Intervention of Frailty in Elderly Patients in China (Geriatric Medicine Branch of Chinese Medical Association et al. 2017) recommends a multidimensional intervention model for acute‐phase rapid recovery wards dedicated to older adults, encompassing comprehensive geriatric assessment, nutritional support, medication management, and individualized discharge planning. This model has been empirically validated to significantly improve clinical outcomes in frail older adults (Geriatric Medicine Branch of Chinese Medical Association et al. 2017). Guided by evidence‐based principles, the emergency frailty management protocol proposed in this study integrates six core components: screening for frailty‐related risk factors, development of personalized frailty care plans, medication management, exercise intervention, nutritional intervention and implementation of discharge planning.

#### 4.4.1 Influencing Factors of Frailty

The influencing factors summarized in this study are specific to elderly patients in the ED, which are confirmed by the included studies to be closely related to the frailty status of ED elderly patients. Frailty severity, physical function and physiological reserve in older adults are determined by multidimensional factors (Walston 2018). The onset and progression of frailty are modulated by a combination of physiological and non‐physiological determinants: (1) Physiological factors include involuntary weight loss (specifically ≥ 5 kg within the past 12 months), urinary incontinence, sensory impairment (visual or auditory) and chronic pain; (2) Non‐physiological factors include polypharmacy, delirium, cognitive impairment, dementia, recurrent ED visits or hospitalizations, declined functional status and mobility, recent falls, increased susceptibility to adverse drug reactions, and inadequate social support. A prospective study (Morley et al. 2013) demonstrated that screening for reversible causes of frailty and delivering targeted interventions can significantly improve the prognosis of frail older adults.

#### 4.4.2 Development of Individualized Frailty Management Protocols

Clinical observations have identified a substantial discrepancy between the actual health needs of frail older adults and the service capacity of existing healthcare systems (Feng et al. 2023). The International Clinical Practice Guidelines for Frailty Identification and Management (Dent et al. 2019) emphasize that multidimensional frailty management plans should be developed via shared decision‐making—based on comprehensive assessments of frail older adults' needs—to address unmet care gaps. Aurélie et al. (Kolle et al. 2023) conducted a systematic review of frailty intervention studies and concluded that individualized intervention strategies, tailored to the heterogeneity of frail older adults, yield significantly superior clinical outcomes compared with standardized group interventions.

#### 4.4.3 Medication Management

Given the high prevalence of polypharmacy among older adults (up to 60% in community‐dwelling frail populations (Morley et al. 2013)), medication management is a pivotal component of emergency care for this vulnerable group. Current guidelines (Dent et al. 2019) explicitly state that pharmacological treatment targeting frailty itself is not recommended; instead, MDTs should collaborate with patients and primary caregivers to conduct medication reconciliation, dose optimization, and therapeutic monitoring—interventions proven to enhance medication safety and improve treatment adherence (Geriatric Medicine Branch of Chinese Medical Association et al. 2017).

#### 4.4.4 Exercise Intervention

Regular physical activity is recognized as a core intervention for preserving and enhancing muscle strength, physical function, and mobility in frail older adults (Dent et al. 2017). A multicenter Asia‐Pacific cohort study (Matchar et al. 2017) confirmed that comprehensive exercise interventions—integrating resistance training, balance training, and gait training—reduce fall rates by 32% in older adults discharged from EDs. Given the time constraints and clinical acuity of ED settings, clinical practice should prioritize developing individualized exercise prescriptions based on patients' functional status (assessed via tools such as the Timed Up and Go test) and activity capacity, with progressive loading regimens that balance safety and intervention efficacy.

#### 4.4.5 Nutritional Intervention

As a foundational component of frailty intervention, nutritional intervention remains the most widely implemented strategy for frail older adults in clinical practice (Kolle et al. 2023). Evidence indicates that targeted nutritional interventions can reverse involuntary weight loss and reduce mortality by 28% in malnourished frail older adults (Geriatric Medicine Branch of Chinese Medical Association et al. 2017). These interventions primarily involve two modalities: (1) dietary supplementation (e.g., oral nutritional supplements with high protein content, 1.2–1.5 g/kg body weight/day) and (2) nutrition education focused on healthy food selection. Recent randomized controlled trials (Bombard et al. 2018; Krist et al. 2017) demonstrate that participatory decision‐making in nutrition education significantly enhances older adults' autonomy, improves dietary self‐efficacy, and strengthens food selection capabilities. Nutrition therapists should develop personalized support plans—combining nutritional/protein‐energy supplementation with exercise prescriptions—to deliver integrated nutrition‐exercise interventions. For patients with involuntary weight loss, etiological screening (e.g., ruling out malignancy and gastrointestinal disorders) and targeted nutritional fortification should be prioritized (Kolle et al. 2023).

#### 4.4.6 Discharge Planning

Studies confirm that frailty management education for patients and caregivers reduces the incidence of frailty by 40% and slows its progression by 25% (Dent et al. 2019). Intervention strategies should be stratified by patient acuity:(1) Non‐hospitalized patients (no emergency treatment required): MDTs should provide basic nursing guidance and coordinate community resources (e.g., home care services, community nutritional support) to ensure smooth discharge and timely follow‐up (scheduled within 72 h post‐discharge);(2) Hospitalized or transferred patients: Standardized nursing handovers should document ED care processes, medication adjustments, and subsequent care needs (e.g., rehabilitation goals, nutritional requirements) to ensure care continuity.

Additionally, all frail older adults should receive personalized lifestyle guidance (e.g., sleep hygiene, social engagement) and exercise prescriptions to promote functional recovery. This integrated care model not only emphasizes acute‐phase medical interventions but also establishes a seamless care continuum from the ED to community support, thereby enabling holistic frailty management.

### 4.5 Strengthening Capacity Building for Geriatric Frailty Emergency Management and Optimizing the Elderly‐Centric Environment

To ensure the standardized and sustainable management of geriatric frailty in emergency care, the following strategies are proposed: (1) Develop a continuous professional development program for emergency department personnel, focusing on training in standardized geriatric frailty management protocols and core competencies; (2) Introduce age‐friendly modifications to emergency care settings, encompassing the development of barrier‐free infrastructure, optimization of environmental safety measures and provision of assistive devices, to address the specific needs of elderly patients. This integrated approach—combining workforce capacity building with environmental optimization—can effectively enhance the quality and efficiency of geriatric frailty management in emergency care contexts.

## 5 Limitations

While this study comprehensively synthesizes evidence on geriatric frailty management in emergency care, variations in ethnicity, region, and cultural background may constrain the generalizability of its findings. A key limitation is that this research exclusively included English and Chinese publications, excluding literature in other languages. Future studies should systematically expand and update the evidence base, and rigorously evaluate its feasibility, applicability, clinical relevance and efficacy—with the aim of facilitating the effective translation of evidence into clinical practice and optimizing nursing care quality.

## 6 Conclusion

In conclusion, this study synthesizes 38 high‐quality evidence‐based guidelines on geriatric frailty management from domestic and international sources. These findings advance the understanding of emergency healthcare professionals, family caregivers and patients regarding frailty management, thereby providing actionable guidance for clinical practice. When implementing evidence‐based interventions, healthcare providers should conduct comprehensive assessments tailored to emergency care settings and the individual characteristics of older adults—an approach that enables the development of personalized clinical strategies to foster healthy, active aging in this population.

## Author Contributions

Meiling Yuan, Haipeng Yang and Xia Liu were responsible for the study conception, design, evidence analysis and summary and writing the manuscript. Meiling Yuan, Yueguang Dai and Xiaotong Sun performed the literature searching. Meiling Yuan, Xiaotong Sun and Yuchen Zhang evaluated the quality of literature. All authors discussed the results and contributed to the final manuscript.

## Funding

This work was supported by the Natural Science Foundation of Shandong Province, China (Grant ZR2022MH022).

## Disclosure

Generative Artificial Intelligence Disclosure: OpenAI ChatGPT (GPT‐5.6 Thinking; accessed 12 July 2026) was used to assist with language editing and restructuring in response to editorial and peer‐review comments. The authors reviewed and verified all revisions and remain fully responsible for the accuracy, integrity, and conclusions of the manuscript.

## Conflicts of Interest

The authors declare no conflicts of interest.

## Supporting information


**Data S1:** nop270720‐sup‐0001‐Supinfo1.docx.

## Data Availability

All data supporting the results are derived from publicly available literature and databases, which have been cited in the references.
