# Supplementary material for: Best Evidence Summary for the Management of Frailty in Elderly Patients in Emergency Department–Integrative Review
Source: Nurs Open. 2026 Jul 29;13(8):e70720. doi: 10.1002/nop2.70720 (PMC13420300; doi:10.1002/nop2.70720)
Supplement: Supplementary file 1 — Data S1: nop270720‐sup‐0001‐Supinfo1.docx. [file NOP2-13-e70720-s001.docx]

PubMed search strategy.

| #1 | (Frailty[MeSH Terms]) OR (Debility[MeSH Terms]) |
| --- | --- |
| #2 | (((((Frailty[Title/Abstract]) OR (Frailties[Title/Abstract])) OR (Frailness[Title/Abstract])) OR (Frailty Syndrome[Title/Abstract])) OR (Debility[Title/Abstract])) OR (Debilities[Title/Abstract])) |
| #3 | #1 OR #2 |
| #4 | (((Emergency Department[MeSH Terms]) OR (Emergency Service[Title/Abstract])) OR (Emergency Rooms[Title/Abstract])) OR (Emergency[Title/Abstract]) |
| #5 | #3 and #4 |
| #6 | (((((management[Title/Abstract]) OR (nutrition[Title/Abstract])) OR (exercise[Title/Abstract])) OR (Frailty Screening[Title/Abstract])) OR (Frailty Assessment[Title/Abstract])) OR (patient education[Title/Abstract]) |
| #7 | #5 and #6 |
| #8 | (((((((((guideline[MeSH Terms]) OR (consensus[MeSH Terms])) OR (systematic review[MeSH Terms])) OR (summary of evidence[Title/Abstract])) OR (Meta-analysis[Title/Abstract])) OR (Best practice[Title/Abstract])) OR (Expert consensus[Title/Abstract])) OR (Recommendation[Title/Abstract])) OR (Clinical practice[Title/Abstract])) OR (Clinical decision[Title/Abstract]) |
| #9 | #7 and #8 |
